# Physicochemical evaluation of sausages prepared by lantern fish (*Benthosema pterotum*) protein isolate

**DOI:** 10.1002/fsn3.583

**Published:** 2018-02-15

**Authors:** Marzieh Moosavi‐Nasab, Rezvan Mohammadi, Najme Oliyaei

**Affiliations:** ^1^ Seafood Processing Research Group School of Agriculture Shiraz University Shiraz Iran; ^2^ Department of Food Science and Technology School of Agriculture Shiraz University Shiraz Iran

**Keywords:** Lantern fish protein isolate, pH‐shift process, physicochemical properties, sausage

## Abstract

The purpose of this study was to show how sausages produced with lantern fish (*Benthosema pterotum*) protein isolate at two levels 4% (sample A) and 2% (sample B), and then, the physicochemical and sensory properties of sausages (A and B) were determined during the storage (14, 30, and 60 days) at 4°C. Firstly, fish protein isolate (FPI) prepared at alkaline pH (12). It was found that the acidic value, peroxide value (PV), and thiobarbituric acid (TBA) value of sausages increased during the storage. The highest level of TBA value was found at the second month of storage as 0.51 mg malonaldehyde/kg. The PV and acidic value reached to 9.45 meq/kg and 4.82 at the end of storage, respectively. Moreover, the stiffness, adherence, and springiness were assessed by texture profile analysis which was found sausages containing FPI had stronger texture and structure compared to control sample. The SDS–PAGE analysis identified the proteins from 15.4 to 202.3 kDa, which exhibit no major differences in protein patterns of two types of sausages. Furthermore, sensory evaluation diagnostic was carried out in terms of the sensory attributes such as texture, flavor, odor, color, and overall acceptability, and the results indicated the greatest overall acceptability in samples containing 2% FPI.

## 1 INTRODUCTION

The global increase in meat product supply has strengthened the demand for manufacturing of safe, nutritious, and innovative ones (Yousefi & Moosavi‐Nasab, 2014). Furthermore, the preference of the consumers is directed toward the development of meat products like sausages using alternative sources of protein such as fish and poultry which possess health beneficial effects (Cardoso, Mendes, & Nunes, 2008; Cegielska‐Radziejewska & Pikul, 2004; Conte‐Junior et al., 2010; Lerasle et al., 2014; López‐Caballero, Góamez‐Guillén, Pérez‐Mateos, & Montero, 2005; Panpipat & Yongsawatdigul, 2008; Rahman, Al‐Waili, Guizani, & Kasapis, 2007). Moreover, by increasing awareness about the endless of marine resources, lots of efforts are ongoing to better and more value‐added utilization of the small underutilized fish and the by‐products from the fishing industries (Nolsøe & Undeland, 2009).

The development of restructured fish products from low‐value species as new food ingredient leading to variety of seafood products besides the providing of alternative source of high nutritive protein for reaching the health‐conscious consumers and reduction of environmental problems causing by marine industry (Cardoso et al., 2008; Martins, Costa, Silveira, Brandelli, & Prentice, 2011; Taskaya & Jaczynski, 2009). In addition, one method to avoid overfishing problem and also increase annual fish consumption is using novel procedures and different fish species for generation of alternative products for instance surimi, minced fish, and fish protein isolate (FPI) as a source of protein (Shaviklo, 2006). Thus, lots of researches have been carried out about isolation and utilization of marine proteins in the last years (Chen & Jaczynski, 2007; He, Franco, & Zhang, 2013). FPI produced from different parts of raw material, without retaining the original form of muscle, is usually used as ingredient for the manufacture of value‐added products and gains increased interest in food industries (Foh, Wenshui, Amadou, & Jiang, 2012). FPI has high functional properties especially foaming and emulsifying capacity that causes the physicochemical interaction with other components of food Oliyaei et al., 2017.

FPI prepared by pH‐shift technique has been shown as the promising starting material with better physical properties of film with negligible discoloration because of lower heme protein and lipid contents and also effective elimination of unwanted compounds abundant in minces from dark muscle fish (Arfat, Benjakul, Prodpran, & Osako, 2014). The pH‐shift protein isolation is a novel method based upon differences in solubility that muscle proteins in water exhibit at different pH values. In this process which also called isoelectric solubilization/precipitation method, most muscle proteins are solubilized at high pHs (extreme acidic or alkaline condition) and then insolubilized close to the isoelectric point (pI) of the muscle proteins because of minimum protein charge. Compared with conventional surimi method, it has advantages such as higher yield, high quality, and efficient elimination of insoluble impurities. In pH‐induced protein solubilization method, minced raw materials can be directly subjected to FPI as all contaminating materials with a density different from the proteins can be removed by gravity through centrifugation. Moreover, extraction of sarcoplasmic proteins in this process led to increase in the protein yield (Gehring, Gigliotti, Moritz, Tou, & Jaczynski, 2011; Oliyaei, Ghorbani, Moosavi‐Nasab, Sadeghimahoonak, & Maghsoudloo, 2017; Park & Park, 2007). In this manner, various research efforts were made to characterize the functional properties of FPI by pH‐shift method such as tilapia (Ingadottir, 2004), Atlantic croaker (Kristinsson & Liang, 2006), rainbow trout (Chen & Jaczynski, 2007), channel catfish (Davenport & Kristinsson, 2011), and lantern fish (Oliyaei et al., 2017).

Lantern fishes are abundant group of small fishes (2–15 cm) from Myctophidae family, which live in mesopelagic region and distribute all over the world oceans (Chai et al., 2012). Due to the reduction in other reserves of commercial fishes, this species for human consumption has been considered. However, to the best of our knowledge, there is no research conducted on the utilization of lantern fish protein isolate in the production of sausage, and such information would support the food industry in the use of this new substrate for sausages.

In this work, the FPI from lantern fish (*Benthosema pterotum*) was extracted with alkaline pH‐shift method (pH 12) and then sausages manufactured with FPI at two levels. The main objective of this study was to produce fortified sausage with FPI as novel meat‐based product and to evaluate physicochemical and sensorial characteristics of sausages prepared by FPI compared with samples produced with soy protein.

## 2 MATERIALS AND METHODS

### 2.1 Raw materials

Lantern fish (*B. pterotum*) were obtained from the Oman Sea (south of Iran) and upon arrival in our laboratory (Seafood Processing Research Group, Department of Food Science and Technology, Shiraz University, Iran), stored at −18°C until use.

### 2.2 Fish protein extraction

The alkaline pH‐shift protein isolation process was applied according the method described by (Davenport & Kristinsson, 2011) with slight modification. Frozen lantern fish, approximately 1 kg, was thawed overnight in the refrigerator (4°C) and then minced. The minced fish slurry was homogenized (homogenizer IKA T25D) after mixed with distilled water (1:6 w/v), and the pH was adjusted to 12 using 0.5 N NaOH, and then, the solution was stirred gently for an hour. The homogenate was centrifuged at 8,500 × g in precooled (4°C) for 10 min and the upper layer collected. The supernatant was adjusted to pH 5 (pI) using 0.5 N HCl and allowed to stand for precipitation of proteins. After readjustment, the solution was centrifuged at 8,500 × g for 10 min at 4°C. Finally, the bottom sediment layer was collected and freeze‐dried. The sample was milled and stored at 4°C until use.

### 2.3 Sausage production by fish protein isolates

Table 1 reveals the basic formulation of the sausages. The sausages formulated in two groups A and B containing 4% and 2% FPI, respectively. C was a control sample without FPI. Each sausage composed 40% minced meat, 13.5% oil, 8% starch, 3% wheat flour, 3.4% gluten, and 1.6% salt. Materials were weighed and at 4°C for 2 min homogenized using cutter. The sausages were cooked at 82°C for an hour and a half baking operation was carried out and cooled with cold water up to 20°C and then maintained in the fridge. Control sample (C) was produced with soy protein isolate that was used instead of FPI.

**Table 1:** Base formulation of sausages

Constituents	A	B	C
	Amount (%)	Amount (%)	Amount (%)
Minced meat	40	40	40
Sunflower oil	13.50	13.50	13.50
Soy protein	0	2	4
FPI	4	2	0
Gluten	3.40	3.40	3.40
Starch	8	8	8
Salt	1.60	1.60	1.60
Sodium polyphosphate	0.30	0.30	0.30
Nitrite	0.012	0.012	0.012
Ascorbic acid	0.02	0.02	0.02
Spices	0.83	0.83	0.83
Wheat flour	3	3	3
Milk powder	1	1	1
Crush ice	24.23	24.23	24.23

### 2.4 Chemical composition

Determination of sausage composition (moisture, lipid, protein, ash) was carried out according to AOAC (2005). Moisture content was determined using an oven. Kjeldahl and Soxhlet–Henkel methods were used for the determination of total protein (crude protein, *N* = 6.25) and fat content, respectively. Also, ash content was measured by mineralization at 550°C.

### 2.5 Residual nitrite level

Five grams of fined sausages was carefully weighed and homogenized in 40 ml of hot water (80°C) and then stirred within water bath (90°C) for 20 min then followed by cooling at ambient temperature and reached final volume to 500 ml by distilled water; 2.5 ml of sulfanilamide reagent was added to 20 ml filtrated solution. Later, 2.5 ml NED (0.2 g *N*‐(1‐naphthyl) ethylenediamine·2HCl in 150 ml 15% v/v acetic acid solution) was added and raised up to 50 ml. Absorbance was measured at 540 nm. Control sample contained 45 ml distilled water, 2.5 ml sulfanilamide, and 2.5 ml NED (AOAC, 2005).

### 2.6 Acidic value

Acidic value is the mass of KOH or NaOH in milligrams required to neutralize 1 g of sample. According to the method AOCS (1997), first, using solvent, fat was extracted using chloroform and the proportion of lipid into solvent was detected. Twenty‐five milliliters of the filtrate was added to 25 ml ethanol neutralized by NaOH 0.1 N and 0.5 ml phenolphthalein (indicator), followed by stirring completely and standardization with NaOH up to appear stabilize pink color (AOCS, 1997).

(1) $$\text{Acidic value}\;\left(\frac{\text{mg}}{g}\right)=\frac{\left(S-B\right)\times \text{normality of NaOH}\times \;56.1}{\text{weight sample (g)}}$$

S: utilized NaOH for sample (ml);

B: utilized NaOH for control sample (ml).

### 2.7 Peroxide value (PV)

Peroxide value was measured based on AOAC (2005). The sample (3 g) was weighed and heated in a water bath at 60°C for 3 min to melt the fat, and then shaken thoroughly for 3 min. Then, 30 ml of acetic acid–chloroform solution (3:2 v/v) was added to dissolve the fat. After filtration of sample by Whatman filter paper under vacuum, saturated potassium iodide solution (1 ml) was added to the filtrate. Finally, it was titrated with standard sodium thiosulfate (25 g/L). PV was calculated and expressed as milliequivalent peroxide per kg of the sausage sample:

(2) $$\text{PV}\;\left(\frac{\text{meq}}{\text{kg}}\right)=\frac{\left(S\times N\right)}{\text{kg}}\times 100,$$

where *S* is the volume of titration (ml), *N* the normality of sodium thiosulfate solution (*N* = 0.01), and *W* the sample weight (kg).

### 2.8 Thiobarbituric acid (TBA) value

Thiobarbituric acid‐reactive substances assay was performed as described by Mei, McClements, Wu, and Decker (1998) with slight modification. Sausage sample (5 g) was homogenized in 15 ml of distilled water and then mixed (1 ml) with 2 ml of stock solution containing 0.375% TBA, 15% TCA, and 0.25 N HCl. Then, the mixture was heated (15 min in boiling water) to develop a pink color, cooled in tap water, and then centrifuged at 2,000 × g for 15 min. The absorbance of the supernatant was determined spectrophotometrically (model UNICO UV‐2100 Spectrophotometer) at 532 nm. TBA value was expressed as milligrams of malonaldehyde/kilogram of sausage.

### 2.9 Texture profile analysis (TPA)

Texture profile analysis and force at cutting were measured by Texture Analyser (Texture Pro CT V1.3 Buil 15). In these tests, 2‐cm‐thick sausages were prepared and compressed two times by probe (5.2 cm diameter) until the 20% of initial height with constant rate (2 mm/s) is reached. Stiffness (the maximum force in the first stage of compression), adherences (area of positive force pressing the second stage to the first stage), and springiness (distance at which food restores its initial height) were determined (Bourne, 1978).

### 2.10 SDS–polyacrylamide gel electrophoresis (SDS–PAGE)

The pattern of protein was analyzed using SDS–PAGE, as described by Laemmli (1970). Sample (3 g) was mixed with 27 ml of 5% SDS (85°C) and homogenized at a speed of 11,000 rpm for 1 min. The sample was then heated at 85°C for 60 min, followed by centrifugation at 8,000 × g for 5 min at ambient temperature. The supernatants were mixed (1:1 v/v) with a sample buffer (0.5 mol/L Tris–HCl, pH 6.8, containing 4% SDS, 20% glycerol, and 10% BME), heated for 3 min in a boiling water bath, and then loaded (20 μg protein) into the polyacrylamide gel (10% running and 4% stacking gel) assembled in an electrophoresis unit at a constant current of 15 mA per gel. After electrophoresis, the gels were stained using Coomassie Brilliant Blue R‐250 (0.02%) for 2 hr. Destaining was performed using 50% methanol and 7.5% (v/v) acetic acid. The molecular weights of the products of proteolysis were estimated by reference to the relative mobilities of standard proteins.

### 2.11 Sensory evaluation

The sensory quality of the fortified sausages was assessed by 12 trained panelists. The assessors were asked to evaluate the color, odor, flavor, overall acceptability, and texture of products using a five‐point hedonic scale with, 4 indicating “like extremely” and 0 “dislike extremely,” compared with a control product (Tsoukalas, Katsanidis, Marantidou, & Bloukas, 2011).

### 2.12 Statistical analysis

Results were expressed as means ± *SD*. Data were statistically analyzed using SPSS software, and the differences between means were evaluated using Duncan's multiple range test. The *t* test was used to compare the results and determine significant differences at *p* < .05. Three replicates were used in each experiment.

## 3 RESULTS AND DISCUSSION

### 3.1 Chemical composition

The chemical composition of sausages prepared with 4% FPI (sample A) and 2% FPI (sample B) is shown in Table 2. According to the results, the protein content and ash level of products A and B were significantly higher than that of the control sample (C) at the beginning (*p* < .05). Protein levels ranged from 12.04% to 14.78% and ash content varied from 2.93% to 3.43%. However, no significant difference was observed between lipid content of sausage containing FPI and control sample, and observed fat contents were less than 17%. In addition, the results of the moisture content of two products are represented in Table 3. The moisture content was varied from 54.10% to 56.04%. As shown, the moisture content of the fortified sausage samples (A and B) was significantly higher (*p* < .05) than that of control sample at different times (*p* < .05); however, the reduction in moisture during the storage was observed**.**

**Table 2:** Chemical composition of sausages A, B, and C

Sample	A	B	C
Protein (%)	14.78 (±0.15)^a^	13.67 (±0.11)^a^	12.04 (±0.15)^c^
Lipid (%)	16.94 (±0.15)^a^	16.74 (±0.20)^a^	16.80 (±0.16)^a^
Ash (%)	3.43 (±0.03)^a^	3.17 (±0.04)^b^	2.93 (±0.03)^c^

Data are expressed as mean (±*SD*) (*n* = 3). *Means in each column having different letters are significantly different (*p* < .05).

**Table 3:** The moisture content of sausages A, B, and C stored at 4°C

Time (day)	A	B	C
0	54.06 (±0.10)^Aa^	52.80 (±0.10)^Ba^	51.70 (±0.15)^Ca^
14	51.93 (±0.10)^Ab^	50.66 (±0.15)^Bb^	49.20 (±0.15)^Cb^
30	50.66 (±0.15)^Ac^	48.53 (±0.15)^Bc^	47.43 (±0.15)^Cc^
60	48.40 (±0.10)^Ad^	46.6 (±0.10)^Bd^	45.10 (±0.10)^Cd^

Data are expressed as mean (±*SD*) (*n* = 3). Different capital letters in each row and small letters in each column indicate significant differences (*p* < .05).

### 3.2 Residual nitrite level

The residual nitrite level of the produced sausages was obtained at zero and at the end of storage. Analysis of residual nitrite level was carried out (Table 4), indicating a reduction in nitrite content during storage (*p* > .05). Also, there was no significant difference between nitrite content of sausages prepared with FPI and control.

**Table 4:** Nitrite content (ppm) of sausages (A, B, and C) at 4°C

Time (day)	A	B	C
0	11.74 (±3.20)^Aa^	11.72 (±3.20)^Aa^	11.75 (±3.20)^Aa^
60	8.24 (±3.40)^Aa^	8.17 (±3.40)^Aa^	8.28 (±3.40)^Aa^

Data are expressed as mean (±*SD*) (*n* = 3). Different capital letters in each row and small letters in each column indicate significant differences (*p* < .05).

### 3.3 Acidic value

Results for analysis of acidic value of sausages stored in 4°C are given in Table 5. Analysis of acidic value showed no significant difference between fortified samples and control; however, the increasing storage time resulted in an increasing acidic value (*p* < .05). The initial acidic value of all sausages was approximately 2; thereafter, it enhanced and reached around 9 at the end of storage.

**Table 5:** Acidic value of sausages (A, B, and C) at 4°C

Time (day)	A	B	C
0	2.14 (±0.40)^Ad^	2.22 (±0.30)^Ad^	2.52 (±0.40)^Ad^
14	4.00 (±0.32)^Ac^	4.13 (±0.20)^Ac^	4.31 (±0.30)^Ac^
30	6.71 (±0.35)^Ab^	6.61 (±0.30)^Ab^	6.90 (±0.20)^Ab^
60	9.23 (±0.25)^Aa^	9.13 (±0.40)^Aa^	9.45(±0.25)^Aa^

Data are expressed as mean (±*SD*) (*n* = 3). Different capital letters in each row and small letters in each column indicate significant differences (*p* < .05).

### 3.4 Peroxide value

Estimation of primary oxidation products was carried out by PV because lipid oxidation can be occurred during storage as a result of the penetration or intermixing of oxygen in the tissue. According to Table 6, PV of sausages increased statistically during different times (0, 7, 14, 30, and 60 days); however, no significant differences were observed between sausages formulated with FPI and control. As indicated, the PV continuously increased up to 4.71, 4.22, and 4.82 meq/kg in sausages A, B, and C, respectively, for 60 days of storage.

**Table 6:** Peroxide value of sausages (meq/kg) at 4°C

Time (day)	A	B	C
0	1.59 (±0.20)^Ad^	1.42 (±0.35)^Ad^	1.73 (±0.15)^Ac^
14	2.72 (±0.45)^Ac^	2.41 (±0.45)^Ac^	2.86 (±0.50)^Ab^
30	3.94 (±0.50)^Ab^	3.44 (±0.50)^Ab^	2.86(±0.50)^Ab^
60	4.71 (±0.60)^Aa^	4.22 (±0.60)^Aa^	4.82 (±0.60)^Aa^

Data are expressed as mean (±*SD*) (*n* = 3). Different capital letters in each row and small letters in each column indicate significant differences (*p* < .05).

### 3.5 TBA value

The TBA value of sausages prepared with FPI was measured at different times (0, 14, 30, and 60 days). As can be seen (Table 7), increasing levels of FPI increased TBA value of sausages; however, no significant difference was observed between fortified sausages and control. TBA increased after 60 days of storage. Nevertheless, the highest TBA value (0.51 mg malonaldehyde/kg) resulted at the end of storage in the control (Table 7). In addition, the sausages formulated with 4% FPI represented a higher amount of TBA rather than sample containing 2% FPI.

**Table 7:** TBA value sausages (mg malonaldehyde/kg mince) at 4°C

Time (day)	A	B	C
0	0.16 (±0.03)^Ad^	0.15 (±0.02)^Ad^	0.17 (±0.03)^Ad^
14	0.26 (±0.04)^Ac^	0.25 (±0.02)^Ac^	0.27 (±0.02)^Ac^
30	0.39 (±0.03)^Ab^	0.38 (±0.04)^Ab^	0.40 (±0.04)^Ab^
60	0.50 (±0.03)^Aa^	0.49 (±0.04)^Aa^	0.51 (±0.05)^Aa^

Data are expressed as mean (±*SD*) (*n* = 3). Different capital letters in each row and small letters in each column indicate significant differences (*p* < .05).

### 3.6 Texture profile analysis

Texture profiles (stiffness, adherences, and springiness) of sausages prepared with FPI are listed in Table 8. According to the table, addition of FPI improved the texture properties of sausages. The results of texture analysis showed a significant effect of fortification on stiffness. Sample A had higher stiffness parameter rather than other samples (*p* < .05); this parameter increased during storage (*p* < .05). Also, sausages prepared with higher amounts of FPI (sample A) showed higher adherence values than other sausages (*p* < .05). Moreover, during 14 days, adherences decreased significantly but after that no significant difference was observed. In addition, the results of springiness revealed no significant difference even with decline of springiness during storage. Overall, evaluation of TPA indicated that fortification led to stronger texture and structure in sausages. However, the stiffness increased and adherence and springiness decreased during the storage.

**Table 8:** Texture profiles (stiffness, adherences, and springiness) of raw sausages at 4°C

Time (day)	Stiffness			Adherences			Springiness		
	A	B	C	A	B	C	A	B	C
0	2.49 (±0.20)^Ad^	2.16 (±0.20)^Ba^	1.68 (±0.15)^Cd^	0.82 (±0.02)^Aa^	0.78 (±0.01)^Ba^	0.74 (±0.02)^Ca^	0.96 (±0.07)^Aa^	0.93 (±0.04)^Aa^	0.91 (±0.05)^Aa^
14	2.72 (±0.20)^Ac^	2.38 (±0.20)^Bc^	1.87 (±0.10)^Cc^	0.77 (±0.02)^Ab^	0.74 (±0.02)^Bb^	0.69 (±0.02)^Cb^	0.93 (±0.03)^Aa^	0.91 (±0.02)^Aa^	0.91 (±0.04)^Aa^
30	2.93 (±0.15)^Ab^	2.52 (±0.10)^Bb^	2.03 (±0.10)^Cb^	0.77 (±0.02)^Ab^	0.72 (±0.02)^Bb^	0.68 (±0.01)^Cb^	0.92 (±0.05)^Aa^	0.90 (±0.02)^Aa^	0.90 (±0.02)^Aa^
60	3.13 (±0.20)^Aa^	2.66 (±0.10)^Ba^	2.17 (±0.10)^Ca^	0.75 (±0.02)^Ab^	0.70 (±0.02)^Bb^	0.67 (±0.02)^Cb^	0.90 (±0.06)^Aa^	0.90 (±0.02)^Aa^	0.89 (±0.03)^Aa^

Data are expressed as mean (±*SD*) (*n* = 3). Different capital letters in each row and small letters in each column indicate significant differences (*p* < .05).

### 3.7 SDS–PAGE

**Figure 1:**
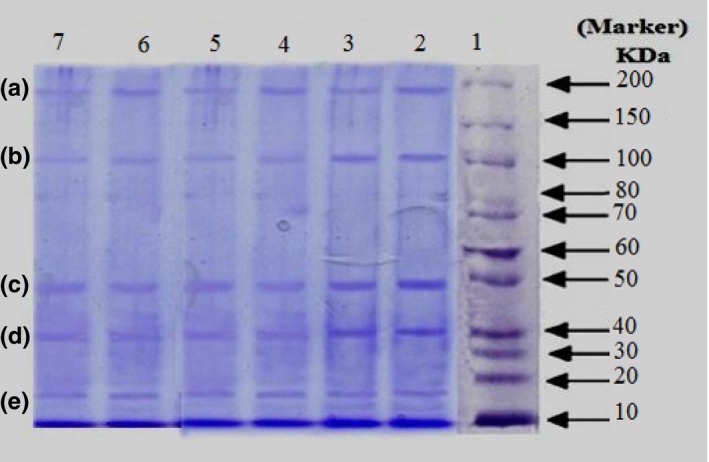
**SDS** **–** **PAGE** **electrophotograms of sausages at zero and day 60 at 4°C. Column 1: marker, columns 2, 4, and 6: samples A, B, and C at zero time, respectively, and column 3, 5, and 7: samples A, B, and C at day 60, respectively**

Figure 1 illustrates SDS–PAGE electrophotograms of sausages prepared with FPI to explore the patterns of molecular weight of proteins during storage at 4°C. According to the figure, the similar protein band patterns were observed in two products. Figure 1 reveals major polypeptide fish protein isolated in samples A and B. The intensity of the polypeptide band decreased over time. The probable identification of MW of proteins gained is shown in Table 9. Polypeptides with estimated molecular weights of 15.4, 19.3, 39.9, 51.6, 100.1, and 202.3 kDa were predominant in samples.

**Table 9:** Molecular weight of proteins and probable identification of bands in SDS–PAGE pattern

Probable identification	MW (kDa)	Band
Myosin heavy chain	202.3	A
α‐Actinin	100.1	B
Actin	51.6	C
β‐Tropomyosin	39.9	D
Myosin light chains	19.3	E
Myosin light chains	15.4	F

### 3.8 Sensory evaluation

Change in the sensory attributes of fortified sausages with different amounts of FPI was studied at different times (0 and 60 days) and is shown in Table 10. Sensory characteristics were measured in terms of color, odor, flavor, texture, and overall acceptability. Sample B was awarded higher scores in sensory parameters, showing sausages prepared by 4% FPI were not favoured by assessors. According to the results, taste and odor after two months remain unchanged; however, there was a significant difference between two products (*p* < .05). In addition, the results indicate that sausages treated with 2% FPI (sample B) were awarded high score value for color and texture compared with other samples (*p* < .05). Overall, sample B had higher score, which might be due to suitable proportion of FPI.

**Table 10:** Sensory evaluation of sausages at different times (0 and 60 days)

Sensory evaluation	Time (day)	A	B	C
Odor	0	2.08 (±0.20)^Ba^	3.00 (±0.20)^Ab^	2.83 (±0.18)^Ac^
	60	2.08 (±0.20)^Ba^	2.91 (±0.30)^Ab^	2.75 (±0.20)^Ac^
Flavor	0	2.16 (±0.10)^Ba^	3.08 (±0.15)^Ab^	2.83 (±0.30)^Ac^
	60	2.08 (±0.10)^Ba^	3.08 (±0.15)^Ab^	2.75 (±0.10)^Ac^
Texture	0	1.87 (±0.10)^Ca^	3.16 (±0.06)^Ab^	2.51 (±0.10)^Bd^
	60	1.75 (±0.10)^Ca^	3.08 (±0.07)^Ac^	2.33 (±0.10)^Bd^
Color	0	2.00 (±0.10)^Ca^	3.08 (±0.20)^Ab^	2.95 (±0.10)^Bc^
	60	1.83 (±0.10)^Ca^	2.94 (±0.10)^Ac^	2.71 (±0.20)^Bd^
General acceptability	0	1.83 (±0.10)^Ca^	2.83 (±0.10)^Ab^	2.70 (±0.10)^Bc^
	60	1.83 (±0.10)^Ca^	2.66 (±0.10)^Ab^	2.53 (±0.10)^Bc^

Data are expressed as mean (±*SD*) (*n* = 3). Different capital letters in each row and small letters in each column indicate significant differences (*p *< .05).

## 4 DISCUSSION

In this study, two percentages of FPI (4 and 2%) that were incorporated in sausages have been evaluated by their physicochemical and quality properties. The chemical composition of fortified sausages exhibited higher amount of protein and ash content rather than that of control. Higher ash content could be related to mineral from protein isolates (Oliyaei et al., 2017). Also, the increase in overall protein content was the result of added FPI. Moreover, there were no significantly differences between lipid content of sausage containing FPI and control sample because the pH‐shift isolation of protein led to the separation of lipid from isolates based on density and solubility differences at extreme alkaline condition (Azadian, Moosavi‐Nasab, & Abedi, 2012; Kristinsson, Theodore, Demir, & Ingadottir, 2005; Oliyaei et al., 2017). Thus, no additional lipid was observed in sausages. Also, fortified sausages with FPI had significantly higher moisture content at the beginning, which is attributed to the FPI used in their formulations. One of the main important properties of FPI is water binding capacity (WBC), which is extremely influenced by pH. Protein's conformation alters as a result of increasing pH and allows to exposure the water binding groups on surface. Thus, increasing the polarity of proteins led to improvement in the WBC (He et al., 2013; Oliyaei et al., 2017), which is observed in sausages manufactured by FPI. Results showed the reduction in nitrite content of samples during the storage which was similar to dry fermented sausages (De Mey et al., 2014). Bozkurt and Erkmen (2004) observed the rapid decrease in nitrite content of Turkish‐style sausage (sucuk) during storage. Nitrite has an important role in curing, stability of red color, specific texture, and pleasant flavor of meat product. Also, nitrite acts as antioxidants and prevents or retards microbial growth, such as *Clostridium botulinum* (Honikel, 2008). Nitrate can be reduced to nitrite by the action of nitrate‐reducing bacteria (Tsoukalas et al., 2011) or reduction of nitrogenous components to nitrite (Bozkurt & Erkmen, 2004). Analysis of acidic value indicated no significant difference between samples, and however increased during the storage which is related to hydrolysis of phospholipids and TAGs or may be due to production of ammonia and biogenic amines as a result of enzymatic activity (Hughes et al., 2002). Moreover, inappropriate condition for pathogens create by acidic pH as a result of acid products. In addition, usually lactic acid bacteria (LAB) are used in fermentation products (Aslim, Yuksekdag, Sarikaya, & Beyatli, 2005), which led to obtain a rapid pH drop of the batter and subsequently prepare safe product as a result of inactivating spoilage microorganisms and preventing their undesirable (Ammor & Mayo, 2007). It seems that fortification had no significant effect on PV value of samples; however, this parameter increased over storage time that was similar to the reports of Tokur, Polat, Beklevik, and Özkütük (2004) which evaluated PV alteration in fish burger obtained from tilapia (*Oreochromis niloticus*). They claimed that PV significantly was enhanced at the beginning and then reached to 5.03 meq/kg at the sixth month and finally reduced to 0.82 meq/kg at the eighth month. Mahmoudzadeh et al. (2010) reported that PV increased significantly in fish burgers prepared from deep flounder (*Pseudorhombus elevatus*) and brushtooth lizardfish (*Saurida undosquamis*) at the end of second month of storage. Moreover, the same results were reported by the Vanitha, Dhanapal, and Reddy (2015) during the refrigerated studies of fish burger from Catla (*Catla catla*). The PV of fresh sample obtained 4.62 meqO_2_/kg and finally reached to 7.28 meqO_2_/kg. TBA value is an important relevant characteristic of meat product that indicates the oxidation state and on later stage rancidity of the product. The results of TBA value of samples showed the continuous increase in TBA value during refrigerated storage of 30 days. A positive relation was found between TBA and acidic value as well as PV, which demonstrated a suitable coincidence between these oxidation factors. This increase might be due to a concomitant increase of oxidation of fatty acids and lipid peroxidation during storage (Nayeem, Chauhan, Khan, Siddiqui, & Sidduqui, 2017). Tokur et al. (2004) gained the same results about tilapia burger over seven months of storage and then suddenly enhanced. In their study, there were some variations in TBA because of its interaction with some biological compounds in fish muscle (Aubourg, Rey‐Mansilla, & Sotelo, 1999). Also, the same trend was observed in Taşkaya, Cakli, Kişla, and Kilinç (2003), which evaluated TBA values of two groups of fish burgers from fresh (A) and frozen‐thawed rainbow trout fillets (B) at 4°C for 21 days. The final results of TBA value of two groups A and B gained 1.38 mg malonaldehyde/kg and 1.00 mg malonaldehyde/kg, respectively. Moreover, TBA level was under the influence of storage time as Bozkurt and Erkmen (2004) observed the increase in TBA values with increasing storage time of Turkish dry fermented sausage. In addition, it seems that the aerobic condition of packaging can accelerate the lipid oxidation of meat products (Byun, Lee, Jo, & Yook, 2001). Oxidative rancidity, which is a type of complex deterioration in fatty fish, was usually detected using the TBA method (Icekson, Drabkin, Aizendorf, & Gelman, 1998). TBA values introduce the secondary oxidized products for instance malondialdehyde and biogenic amine resulting from oxidation (Kurt & Zorba, 2009). However, TBA measurement is not an appropriate method as general references of rancidity, because TBA value is depending on wide range of parameters such as specie, dietary status, age, and raw or cooked meat A (Fernández, Pérez‐Álvarez, & Fernández‐López, 1997). Overall, we can conclude that FPI has no influence on oxidative rancidity as a result of elimination of lipid during alkaline condition of alkaline‐aided process. The TPA results of fortified sausages exhibited the higher texture attribute rather than control, as expected. Addition of FPI caused improvement in stiffness and adherences, although no significant differences were observed in springiness. Moreover, stiffness increased but adherences and springiness decreased during the storage. This resulted from high concentration of myofibril proteins in formulation of A and B, as produce approach caused change in protein conformation and disposal of functional groups (Hultin, Kristinsson, Lanier, & Park, 2005). However, loss of water and subsequently decline of brittleness during storage were the most important result, leading to increase in stiffness (Herrero et al., 2008). The SDS–PAGE illustration of sausages fortified by FPI demonstrated the higher amount of actinin, actin, and β‐tropomyosin bands in samples A and B rather than C which was related to FPI and high concentration of myofibril proteins. Moreover, no decrease in major bands was observed, which indicates the stability of proteins during two months. Furthermore, sarcoplasmic proteins are soluble in water or dilute salt; however, co‐aggregation of some of them with myofibril proteins at isoelectric point may occur during alkaline‐aided process and are thus recovered with the myofibril proteins (Halldórsdóttir et al., 2011; Kristinsson & Ingadottir, 2006). The results are similar to research findings by Foh et al. (2012) and Kristinsson and Liang (2006). Applying FPI affects the sensory attributes of sausages. According to the sensory properties, it can be concluded that most of the negative attributes are related to sample containing 4% FPI and the sausage prepared with 2% FPI had superior attributes rather than others. However, production of some volatile compounds resulting from lipid oxidation and protein decomposition may have undesirable effect on color, odor, flavor, and texture (Tokur et al., 2004), which was the result of low score of sausage with 4% FPI. Orak and Kayisoglu (2008) revealed that sensory properties altered faster than chemical changes over frozen storage. Moreover, the off flavor of samples may be related to WOF (warm‐over flavor). WOF is one of the main reasons of quality loss in processed and frozen meat products and starts with oxidation of meat fats. WOF contains odors and flavors, which usually indicates stale or rancid (Brewer & Decker, 1998).

In addition, off colors may be created by the interaction of different components such as hydrocarbons, ketones, aldehydes, and acids with proteins (Thanonkaew, Benjakul, Visessanguan, & Decker, 2006).

## 5 CONCLUSIONS

The sausage fortified with lantern fish protein isolate is an attempt to develop valuable meat product. Therefore, FPI‐incorporated sausage could be one of the best meat products for consumer. In the present study, the pH‐shift method and precipitation at isoelectric point were used as suitable approach to extraction of protein, and then, different levels of FPI (4 and 2%) were added to sausages, and the physicochemical properties and quality changes of sausages consisting FPI were evaluated during the storage (14, 30, and 60 days) at 4°C. It was found that there were no significant differences between lipid content of fortified sausages with FPI during storage. Storage studies indicated that nitrite content of samples reduced, although acidic value, TBA, and PV enhanced significantly. Moreover, increase in stiffness and decrease in adherence and springing were obtained from TPA results. Also, the stability of protein during two months was suitable and no hydrolysis occurred. In addition, lantern fish protein isolate‐added sausage had improvement in sensory attributes compared with control sausage. Such change in a sausage product was greater with the addition of FPI than control. This study demonstrated that an acceptable sausage can be made with 2% FPI, which can improve sensorial properties of product with concurrent enhancement of their nutritional value. It was noticed that appropriate percentage of FPI is important to produce good quality product, and the addition of FPI is useful in preparing a sausage.
